# Enhanced Polonium Concentrations in Aerosols from the Gulf Oil Producing Region and the Role of Microorganisms

**DOI:** 10.3390/ijerph182413309

**Published:** 2021-12-17

**Authors:** Montaha Behbehani, Fernando Piedade Carvalho, Saif Uddin, Nazima Habibi

**Affiliations:** 1 Environment and Life Sciences Research Center, Kuwait Institute for Scientific Research, Safat 13109, Kuwait; mbahbaha@kisr.edu.kw (M.B.); nhabibi@kisr.edu.kw (N.H.); 2 Instituto Superior Tecnico, Campus Tecnologico e Nuclear, 2695-066 Bobadela-LRS, Portugal; carvalho@ctn.tecnico.ulisboa.pt

**Keywords:** aerosol, bioaerosol, enhanced radioactivity, bioconcentration, polonium, lead

## Abstract

This study provides the first data set of ^210^Po and ^210^Pb activity concentrations in the organic and inorganic components of several particle size classes of aerosols collected at two sampling stations in Kuwait. The ^210^Po concentrations in the aerosols (Bq/g) were similar in all of the particle size classes, but as most (91%) of the aerosol load was made of fine fraction particles of PM_0.39–2.5_ _µm_, most of the ^210^Po activity was carried by this aerosol fraction. At the two sampling stations, the ^210^Po/^210^Pb activity concentration ratios in the aerosols were similar, stable around the year, and averaged 1.5 (range 1.2–1.9), much higher than the typical activity concentration ratios of these radionuclides in unmodified (background) aerosols, with Po/Pb < 0.1. The aerosol enrichment in ^210^Po was likely originated from the oil industry, specifically by gas flaring and oil refining in the Gulf region. Radionuclide analysis in the organic and inorganic components of aerosols showed that the ^210^Po concentration in the organic component was one order of magnitude higher than the ^210^Po concentration in the inorganic component, in contrast with ^210^Pb, which displayed similar concentrations in both organic and inorganic aerosol components. The ^210^Po carrying organic component of aerosols was investigated and it was found to be largely composed of microorganisms with high microbial and fungi diversity, with the phyla *Proteobacteria*, *Ascomycota*, and *Basidiomycota* being dominant among the bacteria and with *Zygomycota* being dominant among the fungi. Therefore, we are facing an active concentration process of the atmospheric ^210^Po carried out by microorganisms, which underlies the ^210^Po enrichment process in the organic component of aerosols. This bioconcentration of polonium in bioaerosols was unknown.

## 1. Introduction

Polonium (^210^Po, T_1/2_ = 138.4 d) and radioactive lead (^210^Pb, T_1/2_ = 22.3 a) are naturally occurring radionuclides that belong to the uranium radioactive decay series. Under the environmental conditions that prevail at the surface of our planet, both radionuclides exist in the solid state, and their presence in the atmosphere is primarily related to the radioactive decay of airborne radon gas (^222^Rn; T_1/2_ = 3.8 d) [1]. In contrast to radon, which is a noble gas with atoms that carry a neutral electric charge, all of the radon daughters are positive ions that are highly particle reactive and, once formed, they become rapidly attached to particles. In the atmosphere, polonium-210 ions become attached to aerosol particles within 40 to 180 s after its formation from the radioactive decay of the precursor radon daughters [2]. Dry and wet atmospheric depositions continuously scavenge radon daughter ions from the atmosphere, preventing the formation of a secular radioactive equilibrium between radon and radon progeny and maintaining a very significant radioactive disequilibrium between ^210^Pb and ^210^Po, with ^210^Po/^210^Pb ratios that are generally much lower than 0.1 [3].

Since the pioneering work by Poet and Martell [4], the radioactive disequilibrium ^210^Po/^210^Pb and the disequilibria between ^210^Po/^210^Bi/^210^Pb have been used as tracers of atmospheric aerosols, and, in particular, to determine the mean residence time of aerosol particles in the troposphere [3,5]. Later research did elucidate that the ^210^Pb and ^210^Po in the atmosphere may have origins other than the radioactive decay of atmospheric radon. Volcanic emissions, atmospheric emissions from certain industrial facilities, forest fires, coal burning, and nuclear weapons tests have been identified as sources of ^210^Po and sometimes of ^210^Pb that have been released into the atmosphere [6,7,8,9,10,11,12]. The particularity of these additional sources, which generally generate strong inputs of ^210^Po with minor or no ^210^Pb input, is the increase in the ^210^Po/^210^Pb ratios of the naturally occurring radionuclides in the background aerosols to values that are much above 0.1 and that may even exceed 10 [9,12].

From surface air, the particle attached ^210^Po and ^210^Pb can easily be inhaled, contributing to the radiation exposure of lungs. Polonium-210 and lead-210 are also present in foods and are ingested with the diet [13,14,15]. The intake of these radionuclides through the inhalation and ingestion pathways originates an internal radiation dose to human beings that is generally higher than the dose from other naturally occurring radionuclides, with the exception of radon [16]. The activity-to-dose conversion factors are 4.3 µSv/Bq for inhaled ^210^Po and 1.2 µSv/Bq for ingested ^210^Po, and 5.6 µSv/Bq and 0.69 µSv/Bq for inhaled and ingested ^210^Pb, respectively. These values are much above the conversion factors for other naturally occurring radionuclides [17]. With such high conversion factor values, additional exposures to enhanced ^210^Po activity in the environment may significantly increase the radiation dose received by human beings. Therefore, research on the environmental behavior of ^210^Po and ^210^Pb and ^210^Po-^210^Pb environmental monitoring are highly valued for assessing radiation exposure and ensuring the radiation protection of the public [18,19].

Studies on the behavior of ^210^Po and ^210^Pb in the air have been conducted at several locations [2,3,9,10,11,20,21,22,23,24,25,26,27,28]. Some of these studies have reported ^210^Po/^210^Pb concentration ratios that are occasionally much above the unity, and such exceptionally high ratios have generally been associated with ^210^Po releases from anthropogenic sources involving high temperatures, such as metal smelters, ceramic kilns, incinerators, and forest fires [2,3,12,20,21,29,30]. Furthermore, radionuclide analysis in fractionated aerosols has indicated that most of the polonium activity is associated with fine and ultrafine aerosol particles. For example, in Japan over, 70% of the ^210^Po activity in aerosols was reported to be associated with the aerosol size fraction of <0.7 µm [31]. In Poland, 82% of ^210^Po found in aerosols was measured to be within the particle size class fraction 0.1–0.3 µm while 8 to 30% of ^210^Po was measured to be in the size class fraction of less than 0.1 µm and was mostly attributed to emissions from industrial sources [20]. In Portugal, a study on smoke from vegetation and forest fires concluded that most of the ^210^Po in the aerosol was attached to particles that were <1 µm in size [10,11].

Due to the limited radioactivity monitoring of the atmosphere and, in particular, to scarce research on the natural radionuclides ^210^Po and ^210^Pb in the Gulf, few data sets on the levels of these radionuclides in that region are available [18]. Notwithstanding, relatively high levels of ^210^Po have been reported in aerosols across Kuwait, with the largest fraction of activity values being determined in the fine fraction of aerosol particulates (0.39–2.5 µm size), generally confirming the patterns found elsewhere [32].

Aerosol particulates may include inorganic materials, such as soil dust re-suspended by winds and organic materials, such as hydrocarbons, microbes, and pollen. Biomass and fossil fuel burning are considered the two most important sources of primary organic aerosol particles, while secondary organic particles may originate from smog and combustible particles. A significant fraction of organic materials have also been reported to consist of living matter, such as bacteria, fungi, pollen, and viruses, which are also called bioaerosols [33,34,35,36,37]. However, the role of organic matter and especially the role of microorganisms in aerosols in the bonding of airborne radionuclides is totally unknown.

This study focused on polonium (^210^Po) and radioactive lead (^210^Pb) distribution in several particle size classes of atmospheric aerosols and, in each class, their partitioning between the organic and inorganic components, both in an urban environment and in a remote location in the desert of Kuwait. Furthermore, this study addressed the characterization of the organic fraction of aerosols in terms of biological composition.

## 2. Materials and Methods

### 2.1. Aerosol Sampling and Sample Treatment

High-Volume Air Samplers (HVAS) with a six-stage cascade impactor (Tisch Environmental Inc., Cleves, OH, USA) were utilized to collect the aerosol samples. Each sample was a 24 h time-integrated particulate aerosol sample corresponding to the filtration of an average air volume of 815 ± 5 m^3^, passing through the filters with an average flow rate of 0.566 m^3^/min. The air drawn through the cascade impactor allowed the particulates to be trapped on different filters according to the aerodynamic mean diameter of the aerosol particles.

**Figure 1:**
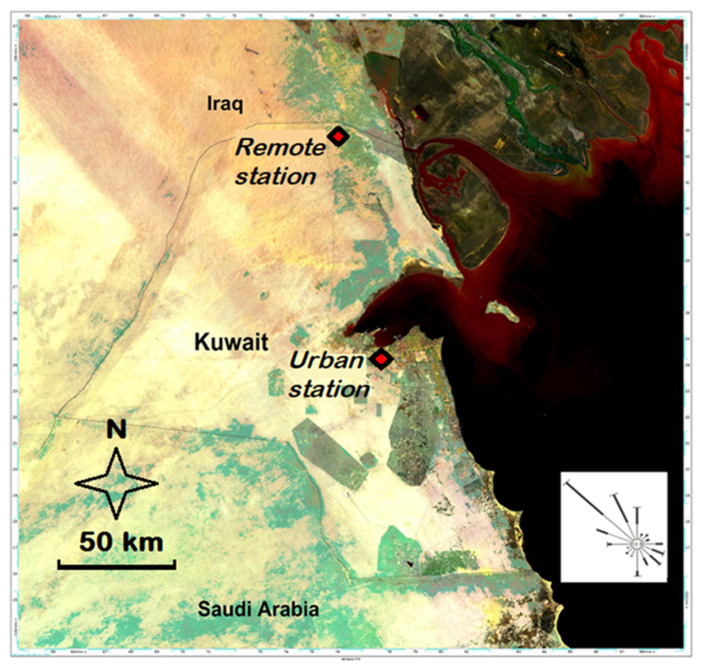
Location of the remote and urban aerosol sampling stations and wind rose for Kuwait City.

Two sampling sites were selected, one near the Kuwait–Iraq border and designated as remote site, and the second within the urban area of Kuwait City (Figure 1). The remote sampling site was located 120 km North of Kuwait City, in an agricultural area in the desert, away and upwind of the urban and industrial sources of potential atmospheric pollutants in Kuwait. The sampling site at Kuwait City was located well within the urban perimeter and in the vicinity of the main commercial port and 15 km east of a major power and desalination plant, but not near industrial sources of atmospheric pollutants, most of which are located south of the city. At both sites, winds predominantly come from the NW all year round.

At each of these sampling sites, two samplers were deployed simultaneously to obtain parallel (or replicate) samples, one for determination of ^210^Po and ^210^Pb and the other for characterization of the microorganisms in the aerosol particulates. A slotted quartz fiber filter (QFF) TE-230 QZ from Tisch Environment Inc., Cleves, OH, USA with the dimensions of 13.3 cm × 14 cm (5.25-in × 5.5-in) was used as a collection substrate in each of the six stages of the cascade impactor, and a 20.3 cm × 25.4 cm (8-in × 10-in) glass fiber filter (TE-G653 from Tisch Environment Inc., Cleves, OH, USA) was used as a base filter. The sampling was carried out at least monthly and took place during the period from February 2019 to February 2020, with 14 samples (and the replicates) being produced at each site. Originally, each 24 h sample was composed of six size classes of aerosol particulates but because the mass of the samples collected on the individual filters was very small, it was decided to pool some of the cascade filters stages. The samples were pooled into three classes of particulate size fractions: above 10 µm (PM_>10_), between 2.5 and 10 µm (PM_2.5–10_)_,_ and between 0.39 and 2.5 µm (PM_0.39–2.5_). Hence, 42 samples, 14 for each of the three size class fractions (and replicates), were obtained from each single sampling site. Sampling was avoided on days with extreme dust resuspension (with visibility under 500 m).

Upon collection, the sample filters were stored in separate cleaned aluminum foil, protected in sealed Ziploc^®^ bags, and transported to the laboratory, where the samples were processed on the same day. The dry weights of the filter before and after the deployment were used to quantify the total mass of the aerosol particulates collected. The filters were weighed using a calibrated Mettler Toledo balance, Columbus, OH, USA (precision ±0.0001 g). From each sampling site, one set of samples was used for ^210^Po and ^210^Pb determination, and the replicate set was used for genomic analysis in order to identify the microbial and fungal communities in the aerosols.

### 2.2. Radioanalytical Procedure

Each of the QFFs for radionuclide analysis was thoroughly brushed with a nylon brush into a Petri dish, and the amount of particulate matter obtained was weighed before and after to correct for losses. The material retrieved was used for analysis. The size-fractionated aerosol samples (PM_>10_, PM_2.5–10_, and PM_0.39–2.5_) were treated to separate the organic and inorganic components, and ^210^Po and ^210^Pb were analyzed in each component separately.

For the separation of the organic and inorganic aerosol components, the aerosol sample material was soaked with 30% H_2_O_2_ in a 1:3 ratio, and the dissolution of the organic materials (microorganisms) was allowed to proceed for over 24 h [35]. The sample was then filtered through a pre-weighted 0.45 µm nylon filter, and the filter dried at room temperature. The dry weight of the sample retained on the filter was determined gravimetrically and considered to be the inorganic component, while the loss in mass was considered as the weight of the dissolved organic material. Afterwards, the nylon filter with the inorganic deposit was completely digested in 20 mL 69% HNO_3_. Previous tests have shown that treating filters with H_2_O_2_ destroys the microorganisms and dissolves organic molecules. Genomic qPCR tests that were taken of filters with the inorganic fraction were negative, confirming that the large protein molecules had been dissolved, which was in contrast with the non-treated filters where genomic DNA was always present in large amounts. Furthermore, the hydrogen peroxide solution with a neutral pH was determined to be unable to extract ^210^Po and ^210^Pb from inorganic soil and sediment particles.

A known activity of ^209^Po (T_1/2_ = 103 a), a radioactive isotope of polonium that does not exist in the environment, was added to both the organic and inorganic solutions of the aerosol particulates so that it could be used as an internal isotopic tracer for the determination of polonium (^210^Po) recovery yield, which varied between 66–90%. The solutions were evaporated and the residue treated with 69% HNO_3_ to ensure the complete mineralization and oxidation of the sample materials. The solutions were taken again to dryness to eliminate the nitric acid and the residue dissolved in 0.5M HCl followed by the addition of ascorbic acid (50 mg) to reduce the oxidation state of Fe and Mn, and the pH of the solution adjusted to 2. The polonium in the solution was then electroplated onto an Ag planchet at room temperature, and the polonium isotopes determined by alpha spectrometry [38]. An alpha spectrometer Mirion Technologies (Canberra) Inc., Meriden, CT 06450, USA, with eight vacuum chambers equipped with passivated implanted planar silicon (PIPS) detectors with a 450 mm^2^ surface area (Mirion^®^) were used for radiometric analysis. This method has been used in previous studies that have been carried out at KISR [14,15,39,40,41,42,43,44,45,46].

After polonium plating, the sample solution was evaporated and the dry residue dissolved in 9 M HCl. This solution was passed through a DOWEX 1^X^8 ion exchange resin column pre-conditioned with 9 M HCl in order to remove any residual polonium (both ^210^Po and ^209^Po) while also allowing the ^210^Pb to be quantitatively recovered. The eluate fraction containing ^210^Pb was stored in a sealed HDPE bottle for about 6 months, and the in-grown ^210^Po from ^210^Pb radioactive decay, after addition of a new spike of ^209^Po tracer was plated on a new silver disc. The appropriate decay and in-growth corrections were applied while calculating the ^210^Po and ^210^Pb activity in the aerosol sample at the collection date [47].

### 2.3. Radioanalytical Quality Assurance

Control filters were also deployed during each aerosol sampling campaign, but no air was blown through them. These filters were used as blanks for the analysis of ^210^Po, ^210^Pb, and microorganisms. Blank nylon filters, the same type of filters used for the separation of organic and inorganic phases and dissolved with the inorganic fraction, were analyzed for ^210^Po and ^210^Pb, and the minimal ^210^Po-^210^Pb activity from the blank was deducted from the results of aerosol sample analysis.

As part of the analytical quality assurance procedure, a Certified Reference Material (CRM), IAEA 446–Baltic Sea Seaweed, was analyzed with each batch of samples. The results obtained for this CRM, which was analyzed for ^210^Po and for ^210^Pb, varied between 10.1 and 10.5 Bq kg^−1^, with a median value of 10.4 Bq kg^−1^. This result compared very well with the ^210^Pb and ^210^Po (in secular radioactive equilibrium) information value of 10.9 Bq kg^−1^, with a 95% confidence interval from 10.2 to 12.0 Bq kg^−1^, provided in the CRM certificate.

### 2.4. Microbiological Characterization

The replicate aerosol samples were analyzed for microbial assemblages using a tandem set of 20 samples. Genomic DNA was extracted using the Wizard^®^ Genomic DNA Purification kit (Promega, Madison, WI, USA). Standard Illumina primers were used to amplify the V3 and V4 regions of the 16S rRNA gene and ITS1and ITS2 regions for bacterial and fungal population identification, respectively [48]. The reaction was carried out in Veriti Thermal Cyclers (Applied Biosystems, Grand Island, NY, USA), with the initial DNA polymerase activation taking place at 95 °C for 3 min followed by 35 cycles each for 30 s at 95 °C, 55 °C, and 72 °C, and a final extension step at 72 °C for 5 min. A total of 5 ng of PCR product was used for library preparation using the NEBNext Ultra DNA library preparation kit (New England BioLabs, Genopole, France). The prepared library was sequenced in Illumina HiSeq 2500 (San Diego, CA, USA) with 2 × 250 cycle chemistry. Raw reads (data deposited on NCBI SRA: SUB6214874; BioProject: PRJNA561928 (accessions SRR10128759-SRR10128778) and fungal sequences under the SRA: SUB; BioProject: PRJNA561929 (accessions SRR10481399-SRR10481418)) were processed in order to determine the quality (Fastqc), adapter trimming, and chimera removal. Pre-processed reads from all of the samples were pooled and clustered into OTUs based on their sequence similarity using the Uclust program (similarity cutoff = 0.97) available in Quantative Insight Into Microbial Ecology (QIIME) 2.0 software. The QIIME generated *.biome files were exported to the MicrobiomeAnalyst in order to generate the taxonomy plots and to calculate their relative abundances (RA) [49]. The OTUs that had been generated by QIIME were filtered by applying a sample prevalence of 30% and inter-quartile range of 20%.

## 3. Results and Discussion

The aerosol samples collected between February 2019 and February 2020 at the two locations are listed in Table 1, with information on the weight of the particulate material collected in each particulate grain size class and the weight of the organic and inorganic components of the aerosols. As the total air volume sampled was equal at both stations every month, the weights of the aerosol samples can be compared directly. The first feature to be noted is that the aerosol sample weights were similar at both sampling sites, with weights averaging 0.9322 ± 0.5405 g at the remote station and 1.0086 ± 0.5368 g at the Kuwait City station. Regarding the aerosol load (mg/m^3^), the two sampling sites in Kuwait were also not very different from each other. Around the year, the aerosol loads ranged from 0.1690 to 2.4733 mg/m^3^ at the remote site and from 0.3064 to 2.5653 mg/m^3^ at the urban site, with higher values generally observed in summer. The percentage of the organic matter in the aerosols was also comparable, representing 15 ± 3% at the remote station and 13% ± 1% at the Kuwait City station (in dry weight).

At both locations, the weight of the finest particle fraction PM_0.39–2.5_ (organic + inorganic) accounted for most of the aerosol load, with 91 ± 14% at the remote station and 92 ± 4% at the Kuwait City station. The large grain size fraction PM_>10_ at the remote station accounted for 1–45% of the total aerosol load, while at the city, it always accounted for a smaller percentage, varying from 1–9% of the total aerosol load. Therefore, although the contribution of fine particles was consistently high and dominant at both locations, the variation in the aerosol grain size composition was wider at the remote station than at the city, probably due to episodes of desert dust resuspension.

Results for the ^210^Po activity concentrations in the aerosol size classes and the organic and inorganic components are shown in Table 2. There was little inter-month fluctuation in terms of the ^210^Po activity concentration in the aerosols at both stations and, within the experimental uncertainty, the average activity concentrations were not different between locations. The total activity concentrations of ^210^Po in the aerosols in the three particulate size classes at the remote site and at the urban site were similar. The ^210^Po per unit mass in the organic component was also identical in both stations and accounted for 88 ± 1% and 89 ± 1% for the ^210^Po activity concentration in aerosols at the remote and city stations, respectively. A key feature of ^210^Po distribution was the much higher concentration in the organic component compared to the inorganic component.

Results for the ^210^Pb activity concentrations in aerosols are shown in Table 3. At the two sampling stations, the activity concentrations of ^210^Pb in the aerosols were comparable in the three particulate size classes and in both the organic and inorganic components, with a mean organic/inorganic ratio of 1.

Table 4 displays the ^210^Po/^210^Pb activity concentration ratios in the aerosol samples within each size fraction. The main feature is the elevated ^210^Po/^210^Pb ratio in the organic components, averaging 5.72 (5.62–5.88), which is in contrast with the ^210^Po/^210^Pb ratio of 0.73 (0.71–0.74) in the inorganic component, and this was observed across the three different particulate sizes. Globally, at both stations, the ^210^Po/^210^Pb concentration ratio in the organic component of the aerosols was about 8-fold (7.77–8.3) higher than it was in the inorganic component (Table 2).

In the total aerosol samples (all size classes), the annual average ^210^Po/^210^Pb ratio was 1.6 (range 1.2–1.9) and 1.4 (range 1.3–1.5) at the remote and at the urban sites, respectively, and this remained remarkably stable throughout the year (Table 5). These ^210^Po/^210^Pb ratios are clearly much higher than the radionuclide ratios in the background aerosols and, as the ^210^Pb concentrations were consistently low, the results show an enhancement of ^210^Po activity in the atmosphere. These results can be compared with data from the aerosol studies reported for other regions around the world [4,7,9,12,16,23,50,51,52,53,54] (Table 5).

It must be noted that in a given location, the ^210^Po levels in atmospheric aerosols may fluctuate with contributions from natural and anthropogenic sources. The results of ^210^Po alone do not give sufficient information for the identification of ^210^Po activity enhancement and potential ^210^Po sources. For example, at the same station, the ^210^Po levels in the atmosphere (Bq/m^3^) may fluctuate throughout the year simply because of variable amounts of re-suspended soil dust. In this case, the ^210^Po activity concentration in the aerosol (Bq/g) would remain constant over time because the source of the radionuclide is always the soil material, although displaying a fluctuating volume activity concentration (Bq/m^3^). In such a case, in the atmospheric aerosol, there would be no enhancement in the ^210^Po concentration compared to the ^210^Pb concentration.

To detect ^210^Po enhancement, the ^210^Po/^210^Pb ratios in the aerosols also must be determined along with the ^210^Po activity concentration values. The values of the ^210^Po/^210^Pb concentration ratios in the radioactive background levels (with atmospheric radon as the main ^210^Po and ^210^Pb source) are much lower than 0.1. Values of ^210^Po/^210^Pb ratios > 0.1 are already indicative of a ^210^Po source(s) other than atmospheric radon, and the ^210^Po/^210^Pb ratios > 1 are definitively due to a strong ^210^Po contribution from other sources. Often, these sources are related to industrial processes that involve high temperatures causing the volatilization of ^210^Po from combusted materials into the atmosphere. The emission of volatilized ^210^Po, followed by cooling and condensation onto aerosol particulates with the consequent enhancement of the ^210^Po/^210^Pb ratios was described for forest fires, urban waste incinerators, and metal smelters [9,54].

In regions with very high oil and gas refinement and production, such as the Gulf region, the likely sources for ^210^Po enhancement in aerosols reside in atmospheric emissions from gas flaring and crude oil refining. Furthermore, the results obtained were similar at both sampling stations in Kuwait, which indicates that the enhancement of atmospheric ^210^Po is not merely from a local point source but it seems to be an enhancement at a regional scale and is probably related to emissions from the entire oil industry around the Gulf.

The composition of aerosol particulates may include diversified materials from several origins, such as soil dust, pollen grains, fungi, microbes, soot, and black carbon from industrial emissions, among others. The contribution of re-suspended soil particles likely accounts for most of the inorganic particulates in all grain size classes. Assuming that the ^210^Po and ^210^Pb concentrations that are present in soil materials are generally in close secular radioactive equilibrium (Po/Pb~1), then it is likely that the ^210^Po contribution from soil dust to the aerosols would be similar to that of the ^210^Pb concentration as determined in aerosols. The excess of ^210^Po in aerosol particulates, about 1.2–1.9 times more than ^210^Pb, thus indicates additional ^210^Po emissions into the atmosphere. Po-210 released with gas emissions into the atmosphere is rapidly adsorbed onto particles, and potentially into particles of all sizes, primarily by electrostatic forces. The identical percent contribution from the three particle size classes to the total ^210^Po activity concentration in aerosols indeed suggests that this may occur. Some radioactive lead (^210^Pb) might also be present in the industrial emissions released into the atmosphere, as reported for fly ash from coal-fired power plants [30], but ^210^Pb is volatilized at much higher temperatures than ^210^Po, which renders the ^210^Pb contribution from anthropogenic sources to be less than the one for ^210^Po; but also distributed in all particulate size classes.

In the aerosols from Kuwait, it was found that inside each size class while the ^210^Pb concentration was similar in the organic and inorganic components (Table 3), the ^210^Po concentration was consistently much higher in the organic component of aerosols (Table 2). This suggests that after the sorption of ^210^Po ions onto aerosol particulates, there is a mechanism for the re concentration and preferential bonding of ^210^Po to the organic component of aerosols.

From investigations in aquatic environments, it is known that ^210^Po displays a very high partitioning coefficient (Kd) and is rapidly removed from the soluble phase through sorption onto suspended particles (Kd = 10^5^) similarly, or even more efficiently than ^210^Pb (Kd = 10^4^–10^5^), and thus both radionuclides are highly particle reactive [55]. In biological systems, ^210^Po is known to be accumulated in tissues rich in proteins and in sulphur-containing molecules with a minor accumulation in lipids [19]. In the same biological systems the concentration of ^210^Pb is much lower than that of ^210^Po, and it is not particularly concentrated in either proteins or in lipids [56].These features drove the investigation to know more about the organic component of aerosols.

The analysis of aerosol particulates revealed that they were not simply made of organic and mineral particles from soot and soils but, instead, aerosol particulates were very rich in microscopic living forms, mainly bacteria, fungi and virus [57,58,59,60].

**Figure 2:**
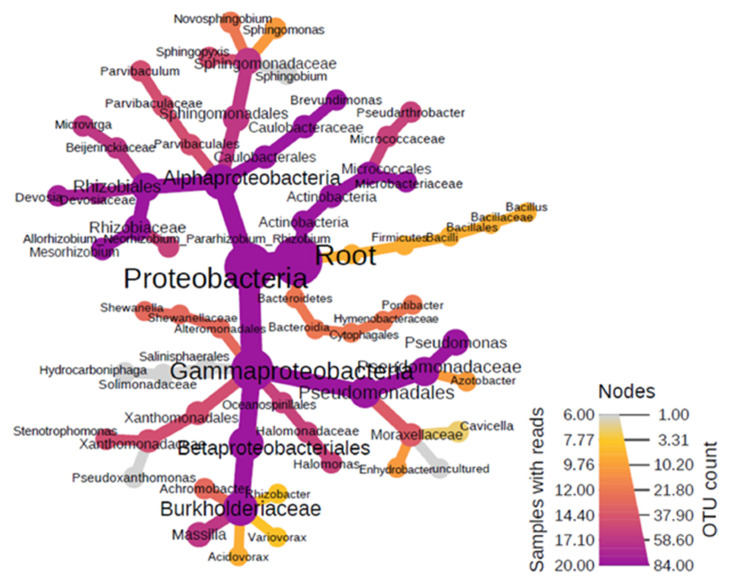
Heat diagram showing dominant bacterial assemblages within the organic aerosol fractions.

The results of the molecular analysis made to characterize the bacteria present in the organic component of aerosols showed dominance of the phylum *Proteobacteria* (RA-97.6%), including the classes *Alpha* (RA-26.5%) and *Gamma-Proteobacteria* (RA-71.0%) (Figure 2). The key orders of *Alphaproteobacteria* were *Caulobacterales* (RA-12.8%), *Rhizobiales* (RA-4.0%), and *Sphingomonadales* (RA-9.3%), finally culminating into the predominant genus of *Brevundimonas* (RA-12.5%), *Allorhizobium* (RA-1.8%), *Devosia* (RA-0.6%), and *Sphingopyxis* (RA-2.7%). The class *Gamma-proteobacteria* was distributed into the main orders of *Pseudomonadales* (RA-23.8%), *Betaprotobacteriales* (RA-43.5%), and *Oceanospiralles* (RA-1.4%). The most prevalent genera in this group were the *Pseudomonas* (RA-2.5%), *Halomonas* (1.4%), *Massilia* (RA-2.4%), and *Stenotrophomonas* (RA-0.32%).

All these bacteria genera belong to both the Gram-negative and Gram-positive bacteria types, and their outer membranes are mainly composed of lipopolysaccharides. It was established that bacteria, especially those involved in sulphur (S) cycle, play a role in the dissolution and mobilization of polonium in rock pore waters and groundwater [61,62] and the ^210^Po concentration by bacteria seems to be a rather general ability of microorganisms. As the S and Po chemical elements are both members of the Group 16 of the Periodic Table and display similar chemical properties, the bacteria will likely process and concentrate polonium ions as they do with sulphur ions.

**Figure 3:**
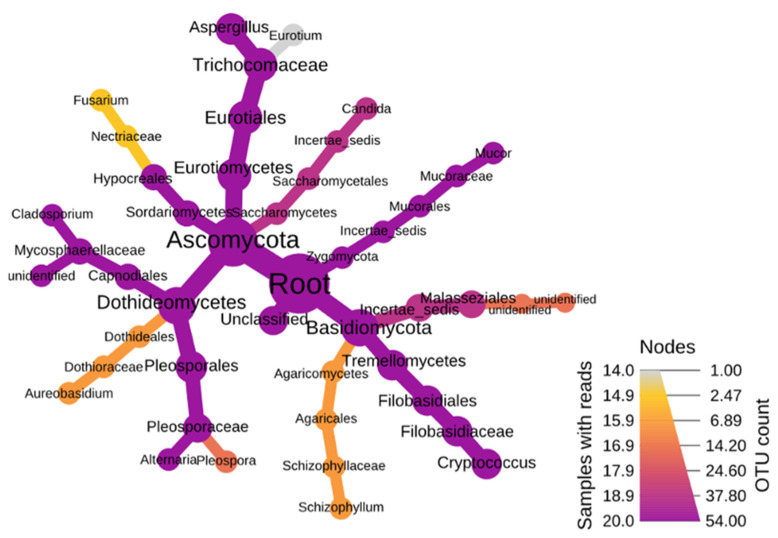
Heat diagram showing dominant fungal assemblages within the organic aerosol fractions.

The fungal phyla were mainly dominated by *Ascomycota* (RA-70%), *Basidiomycota*, and *Zygomycota*. The *Ascomycota* were more diverse compared to the other two phyla. The most dominant classes of *Ascomycota* were *Dothideomycetes* and *Eurotiomycetes* followed by the orders *Capnodiales, Pleosporales*, and *Eurotiales* (Figure 3). These orders culminated into the three major genera of *Aspergillus, Cladosporium*, and *Alternaria*. As for *Zygomycota*, a single genus, *Mucor*, subjugated the branch. In *Basidimycota*, the leading class was *Tremellomycetes*, which terminated into the genus *Cryptococcus*. These results show the presence of a great diversity of fungi in aerosols at both stations and throughout the year.

Fungi have since long been proven to be regulating radionuclide movement in soils [19,63,64,65,66]. No data are available on the bioconcentration of ^210^Po in microscopic fungi, but for macroscopic fungi (mushrooms) as well as for liquens, it is known that they concentrate ^210^Po from the soils and from air [18]. Likewise, the microscopic fungi in aerosols can be expected to concentrate airborne ^210^Po.

**Figure 4:**
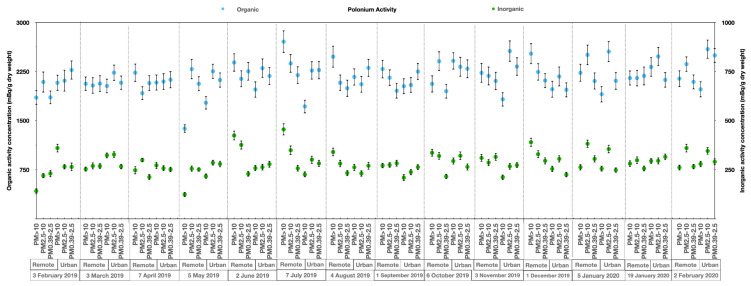
Polonium (^210^Po) concentrations in the organic and inorganic components of different aerosol size fractions at both locations. The samples were collected from February 2019 to February 2020; this ^210^Po study involved 42 samples.

The ^210^Po concentration in the organic and inorganic components within the three aerosol size fractions at two sites is graphically presented in Figure 4. The graphic shows a minor spatial-temporal variability of ^210^Po activity in aerosols, and highlights the higher concentration of ^210^Po in the organic fraction, which was an order of magnitude higher than in the inorganic fraction in all of the three particle size classes and at both sampling sites.

The particulate biological materials in aerosols, or bioaerosols, have not been studied in detail, although they are receiving more attention nowadays due to public health reasons. Bioaerosols contribution to the total aerosol load in the arid climate of Phoenix, USA, was assessed at about 16% to the fine particulate matter PM_>2.5_ and at about 12% to the PM_>10_ on a dry weight basis and these seem to be typical values [67]. These percent values are comparable to the amount of organic components that were determined in aerosols in Kuwait (see above), which indicates that most of the organic material in the aerosols on a wet weight basis were bioaerosols. (Note: assuming a dry:wet weight ratio of 0.05–0.10 for bioaerosols, in an aerosol with 15% dw of bioaerosols, these bioaerosols contribute to approximately 65–78% of the aerosol wet weight).

## 4. Conclusions

Throughout the year, in surface air aerosols in Kuwait the ^210^Po activity is highly enhanced compared to ^210^Pb, with the ^210^Po/^210^Pb ratio in the total particulates averaging 1.5 (range 1.2–1.9). This ratio is in contrast with reports from many regions around the world, where the natural (or near natural) radioactivity background in aerosols displays ^210^Po/^210^Pb ratios < 0.1 (Table 6). In several studies, the ^210^Po enhancement in aerosols was related to ^210^Po emissions from industrial sources, volcanic emissions, and forest fires, thus associated with processes involving high temperatures capable of causing the volatilization of ^210^Po from combusted materials and its release into the atmosphere. This is immediately followed by cooling and ^210^Po condensation onto aerosol particulates. Therefore, the increase in the ^210^Po activity in aerosols with simultaneously high ^210^Po/^210^Pb ratios (>>0.1), such as those observed in Kuwait, implicates the occurrence of a supplementary ^210^Po source with continuous atmospheric emissions.

In this study, the ^210^Po/^210^Pb ratios in aerosols from a remote station in the north of Kuwait and from Kuwait City displayed similar values; thus, the ^210^Po enhancement in the atmospheric aerosols was not the result of local sources in Kuwait City only but was from transboundary origins. This is likely related to the extensive oil production and specifically to gas flaring and oil refining in the Gulf region, and this ^210^Po enhancement in aerosols is nowadays a regional feature, and prevails all year round. In a previous investigation on ^210^Po in the region, one additional sampling station was located south of Kuwait City and was under the influence of emissions from local industry. The ^210^Po concentrations measured there were slightly higher than at the urban sampling site used in this work, indicating that a ^210^Po contribution from local industrial sources also exists [32].

The radionuclide analyses showed that airborne ^210^Po is mostly bound to the organic component of aerosol particulates. The investigation on the composition of organic component of aerosol particulates revealed a great abundance of microscopic fungi and bacteria. Although there is also non-living organic material in aerosols, microorganisms represented about 15% of the dry weight and were estimated to contribute to about 65–78% of the aerosol wet weight. The observed higher ^210^Po concentration in the aerosol organic component, which was in contrast with the ^210^Pb equally distributed between organic and inorganic components, implicates that there is an active ^210^Po concentration by microorganisms in aerosols and not just simple electrostatic adsorption.

We are not aware of any data in the published literature on the partitioning of ^210^Po and ^210^Pb among organic and inorganic components and within size-fractionated aerosols. The concentration of ^210^Po by microorganisms in the aerosols was an unsuspected mechanism, but it efficiently operates the transfer and concentration of atmospheric ^210^Po into the organic/living component of aerosols, which does not happen with ^210^Pb.

The enhanced concentration of ^210^Po in aerosol particulates, especially in the respirable fine fraction of the particulates, may have implications for the radiation exposure of human population. This exposure will be assessed in future work.

## Figures and Tables

**Table 1:** Aerosol samples: weight (in grams) of total aerosol sample and of organic and inorganic components in each particulate size class.

Sampling Date		Remote Site							Urban Site					
	Total Sample(g) ^a^	PM_>10_ (g)		PM_2.5–10_ (g)		PM_0.39–2.5_ (g)		Total Sample(g) ^a^	PM_>10_ (g)		PM_2.5–10_ (g)		PM_0.39–2.5_ (g)	
		Organic	Inorganic	Organic	Inorganic	Organic	Inorganic		Organic	Inorganic	Organic	Inorganic	Organic	Inorganic
3 February 2019	0.7670	0.0027	0.0274	0.003	0.0225	0.106	0.6054	1.0159	0.0061	0.0338	0.0014	0.0092	0.1265	0.8389
3 March 2019	0.9304	0.002	0.0188	0.0025	0.0178	0.1432	0.7461	0.8855	0.0025	0.0245	0.0033	0.0255	0.0979	0.7318
7 April 2019	1.1946	0.0019	0.0207	0.0051	0.0301	0.2194	0.9174	1.4002	0.0024	0.0187	0.0065	0.0445	0.1713	1.1568
5 May 2019	2.0157	0.0032	0.0319	0.0062	0.0372	0.3642	1.573	0.9717	0.0012	0.0116	0.0068	0.0412	0.1293	0.7816
2 June 2019	1.1522	0.0018	0.0189	0.0046	0.0479	0.1252	0.9538	0.2500	0.0009	0.0028	0.0027	0.0183	0.0257	0.1996
7 July 2019	1.9766	0.002	0.0195	0.0085	0.0476	0.3456	1.5534	1.3801	0.0172	0.1039	0.0043	0.0267	0.1302	1.0978
4 August 2019	1.3530	0.0009	0.0111	0.0047	0.0270	0.2422	1.0671	2.0933	0.0047	0.0348	0.0096	0.0554	0.2745	1.7143
1 September 2019	0.5162	0.0059	0.0451	0.001	0.0075	0.0502	0.4065	0.4608	0.0022	0.0129	0.0021	0.0142	0.0614	0.3680
6 October 2019	0.8394	0.0031	0.0167	0.0024	0.0187	0.1254	0.6731	0.8727	0.002	0.0112	0.0092	0.0578	0.088	0.7045
3 November 2019	0.7021	0.0028	0.0141	0.0049	0.0462	0.1027	0.5314	0.9362	0.0094	0.0545	0.0044	0.0364	0.0956	0.7359
1 December 2019	0.1377	0.0074	0.0546	0.003	0.0161	0.0048	0.0518	2.0478	0.0221	0.1372	0.0300	0.2260	0.2057	1.4268
5 January 2020	0.6281	0.0020	0.0061	0.0036	0.0189	0.0747	0.5228	0.8272	0.0067	0.0475	0.0031	0.0278	0.1032	0.6389
19 January 2020	0.5147	0.0008	0.0036	0.0027	0.0153	0.0748	0.4175	0.4841	0.0009	0.0073	0.0016	0.0114	0.0657	0.3972
2 February 2020	0.3226	0.0020	0.0039	0.0006	0.0032	0.0435	0.2694	0.4957	0.0013	0.0093	0.0003	0.0017	0.0729	0.4102
Mean	0.9322	0.0028	0.0209	0.0038	0.0254	0.1444	0.7349	1.0086	0.0057	0.0364	0.0061	0.0426	0.1177	0.8002
SD	0.5405	0.0018	0.0143	0.0020	0.0140	0.1056	0.4279	0.5368	0.0063	0.0379	0.0072	0.0535	0.0620	0.4099
Min	0.1377	0.0008	0.0036	0.0006	0.0032	0.0048	0.0518	0.2500	0.0009	0.0028	0.0003	0.0017	0.0257	0.1996
Max	2.0157	0.0074	0.0546	0.0085	0.0479	0.3642	1.5730	2.0933	0.0221	0.1372	0.0300	0.2260	0.2745	1.7143
Mean ratios:														
Org/Inorg		0.13		0.13		0.18			0.16		0.14		0.15	
Inorg/Org		7.6		7.5		5.7			6.4		7.0		6.8	

^a^ The aerosol particulate concentration (g/m^3^) can be estimated by dividing the aerosol sample weight by 815 m^3^.

**Table 2:** Polonium-210 activity concentrations (mBq/g dry weight ± 1σ) in organic and inorganic components of size fractioned aerosol samples.

Sampling Date	Remote Site						Urban Site					
	PM_>10_		PM_2.5–10_		PM_0.39–2.5_		PM_>10_		PM_2.5–10_		PM_0.39–2.5_	
	Organic	Inorganic	Organic	Inorganic	Organic	Inorganic	Organic	Inorganic	Organic	Inorganic	Organic	Inorganic
3 February 2019	1852 ± 111	142 ± 13	2090 ± 157	220 ± 12	1855 ± 103	231 ± 17	2078 ± 122	360 ± 21	2110 ± 165	265 ± 13	2273 ± 144	264 ± 21
3 March 2019	2062 ± 109	253 ± 12	2036 ± 124	269 ± 17	2065 ± 127	268 ± 14	2029 ± 109	323 ± 14	2232 ± 122	327 ± 16	2078 ± 108	266 ± 13
7 April 2019	2232 ± 138	247 ± 20	1919 ± 102	299 ± 11	2072 ± 119	213 ± 13	2081 ± 121	272 ± 18	2096 ± 125	257 ± 14	2124 ± 129	251 ± 13
5 May 2019	1377 ± 62	124 ± 12	2286 ± 152	255 ± 15	2063 ± 112	251 ± 11	1772 ± 99	217 ± 12	2252 ± 114	285 ± 14	2120 ± 115	279 ± 16
2 June 2019	2388 ± 139	424 ± 24	2138 ± 127	376 ± 22	2253 ± 140	229 ± 13	1974 ± 122	258 ± 15	2300 ± 147	263 ± 16	2181 ± 137	278 ± 16
7 July 2019	2706 ± 170	455 ± 30	2373 ± 140	349 ± 23	2194 ± 134	257 ± 16	1716 ± 101	226 ± 14	2265 ± 138	301 ± 20	2272 ± 139	282 ± 17
4 August 2019	2476 ± 166	340 ± 21	2078 ± 123	281 ± 18	1994 ± 128	233 ± 14	2167 ± 129	262 ± 17	2055 ± 125	231 ± 15	2305 ± 134	270 ± 19
1 September 2019	2288 ± 140	210 ± 13	2155 ± 124	238 ± 13	1955 ± 117	271 ± 16	2025 ± 118	274 ± 16	2044 ± 119	283 ± 15	2249 ± 128	263 ± 14
6 October 2019	2061 ± 128	336 ± 19	2407 ± 147	320 ± 19	1949 ± 109	216 ± 12	2413 ± 130	294 ± 17	2322 ± 148	321 ± 21	2292 ± 142	264 ± 17
3 November 2019	2229 ± 149	310 ± 19	2183 ± 142	286 ± 17	2106 ± 139	315 ± 19	1825 ± 108	212 ± 13	2562 ± 161	267 ± 16	2326 ± 140	273 ± 14
1 December 2019	2523 ± 156	390 ± 22	2243 ± 131	329 ± 19	2112 ± 113	295 ± 17	1979 ± 127	254 ± 15	2174 ± 150	305 ± 18	1971 ± 112	225 ± 13
5 January 2020	2230 ± 134	262 ± 15	2504 ± 155	382 ± 20	2105 ± 128	305 ± 18	1903 ± 122	255 ± 14	2554 ± 158	355 ± 21	2108 ± 133	248 ± 13
19 January 2020	2150 ± 112	281 ± 16	2149 ± 123	298 ± 18	2183 ± 142	256 ± 14	2319 ± 149	294 ± 14	2480 ± 144	295 ± 17	2122 ± 116	315 ± 16
2 February 2020	2141 ± 126	261 ± 14	2362 ± 115	360 ± 21	2092 ± 111	266 ± 13	1978 ± 120	279 ± 15	2591 ± 145	345 ± 18	2496 ± 112	290 ± 17
Mean	2194	288	2209	304	2071	258	2019	270	2288	293	2208	269
SD	308	93	156	49	102	31	186	39	184	34	128	20
Min	1377	124	1919	220	1855	213	1716	212	2044	231	1971	225
Max	2706	455	2504	382	2253	315	2413	360	2591	355	2496	315
Mean ratio Org/Inorg	7.6		7.3		8.0		7.5		7.8		8.2	

**Table 3:** Lead-210 activity concentrations (mBq/g dry weight ± 1σ) in organic and inorganic components of the size fractioned aerosol samples.

Sampling Date	Remote Site						Urban Site					
	PM_>10_		PM_2.5–10_		PM_0.39–2.5_		PM_>10_		PM_2.5–10_		PM_0.39–2.5_	
	Organic	Inorganic	Organic	Inorganic	Organic	Inorganic	Organic	Inorganic	Organic	Inorganic	Organic	Inorganic
3 February 2019	348 ± 21	215 ± 11	303 ± 19	314 ± 17	291 ± 19	324 ± 20	374 ± 21	475 ± 29	378 ± 22	365 ± 23	386 ± 24	367 ± 20
3 March 2019	309 ± 20	365 ± 21	358 ± 23	374 ± 22	328 ± 23	381 ± 22	377 ± 21	446 ± 23	402 ± 22	458 ± 24	374 ± 26	370 ± 23
7 April 2019	391 ± 27	344 ± 18	347 ± 20	374 ± 21	390 ± 24	273 ± 15	368 ± 20	362 ± 20	388 ± 21	347 ± 20	329 ± 17	356 ± 19
5 May 2019	289 ± 19	175 ± 11	386 ± 21	347 ± 18	369 ± 20	307 ± 19	322 ± 16	263 ± 16	376 ± 25	396 ± 27	377 ± 23	379 ± 22
2 June 2019	432 ± 23	581 ± 28	372 ± 21	444 ± 23	412 ± 22	305 ± 18	334 ± 19	358 ± 22	430 ± 19	371 ± 20	375 ± 23	358 ± 18
7 July 2019	403 ± 21	642 ± 32	432 ± 23	429 ± 21	388 ± 18	353 ± 23	304 ± 18	303 ± 18	403 ± 23	427 ± 26	388 ± 22	389 ± 25
4 August 2019	426 ± 22	469 ± 25	384 ± 21	391 ± 23	335 ± 20	305 ± 20	401 ± 22	369 ± 19	374 ± 22	310 ± 19	390 ± 23	384 ± 21
1 September 2019	371 ± 20	283 ± 14	373 ± 22	333 ± 18	354 ± 23	376 ± 16	381 ± 21	378 ± 23	317 ± 15	376 ± 20	423 ± 29	368 ± 19
6 October 2019	402 ± 23	477 ± 29	402 ± 21	454 ± 29	359 ± 19	293 ± 16	432 ± 23	409 ± 25	390 ± 22	433 ± 22	429 ± 21	353 ± 19
3 November 2019	352 ± 16	428 ± 22	360 ± 22	389 ± 20	402 ± 22	434 ± 22	334 ± 22	301 ± 19	438 ± 19	374 ± 22	430 ± 27	377 ± 20
1 December 2019	447 ± 23	546 ± 26	386 ± 16	454 ± 31	367 ± 20	410 ± 18	332 ± 20	358 ± 19	396 ± 24	430 ± 25	359 ± 19	319 ± 19
5 January 2020	330 ± 15	359 ± 20	473 ± 27	512 ± 31	373 ± 20	430 ± 22	337 ± 15	356 ± 20	480 ± 23	483 ± 24	386 ± 20	350 ± 19
19 January 2020	350 ± 22	390 ± 21	380 ± 23	427 ± 25	400 ± 23	364 ± 19	392 ± 22	400 ± 20	417 ± 20	407 ± 21	380 ± 19	403 ± 20
2 February 2020	379 ± 25	368 ± 22	373 ± 21	522 ± 32	387 ± 22	372 ± 20	346 ± 19	388 ± 23	446 ± 24	482 ± 27	464 ± 24	415 ± 21
Mean	374	403	381	412	368	352	360	369	403	404	392	371
SD	45	128	38	60	32	50	34	54	38	49	33	23
Min	289	175	303	314	291	273	304	263	317	310	329	319
Max	447	642	473	522	412	434	432	475	480	483	464	415
Mean ratio Org/Inorg	0.9		0.9		1.0		1.0		1.0		1.1	

**Table 4:** Polonium/lead (^210^Po/^210^Pb) activity concentration ratios in the organic and inorganic components of the size fractionated aerosols.

Sampling Date	Remote Site						Urban Site					
	PM_>10_		PM_2.5–10_		PM_0.39–2.5_		PM_>10_		PM_2.5–10_		PM_0.39–2.5_	
	Organic	Inorganic	Organic	Inorganic	Organic	Inorganic	Organic	Inorganic	Organic	Inorganic	Organic	Inorganic
3 February 2019	5.32	0.66	6.9	0.7	6.37	0.71	5.56	0.76	5.59	0.72	5.88	0.72
3 March 2019	6.67	0.69	5.68	0.72	6.29	0.7	5.38	0.72	5.56	0.71	5.56	0.72
7 April 2019	5.71	0.72	5.52	0.8	5.32	0.78	5.65	0.75	5.41	0.74	6.45	0.7
5 May 2019	4.76	0.71	5.92	0.74	5.59	0.82	5.49	0.83	5.99	0.72	5.62	0.74
2 June 2019	5.52	0.73	5.75	0.85	5.46	0.75	5.92	0.72	5.35	0.71	5.81	0.78
7 July 2019	6.71	0.71	5.49	0.81	5.65	0.73	5.65	0.75	5.62	0.7	5.85	0.72
4 August 2019	5.81	0.72	5.41	0.72	5.95	0.76	5.41	0.71	5.49	0.75	5.92	0.7
1 September 2019	6.17	0.74	5.78	0.71	5.52	0.72	5.32	0.72	6.45	0.75	5.32	0.71
6 October 2019	5.13	0.7	5.99	0.7	5.43	0.74	5.59	0.72	5.95	0.74	5.35	0.75
3 November 2019	6.33	0.72	6.06	0.74	5.24	0.72	5.46	0.7	5.85	0.71	5.41	0.72
1 December 2019	5.65	0.71	5.81	0.72	5.75	0.72	5.95	0.71	5.49	0.71	5.49	0.7
5 January 2020	6.76	0.73	5.29	0.75	5.65	0.71	5.65	0.71	5.32	0.74	5.46	0.71
19 January 2020	6.13	0.72	5.65	0.7	5.46	0.7	5.92	0.74	5.95	0.72	5.59	0.78
2 February 2020	5.65	0.71	6.33	0.69	5.41	0.71	5.71	0.72	5.81	0.71	5.38	0.7
Mean	5.88	0.71	5.83	0.74	5.65	0.73	5.62	0.73	5.70	0.72	5.65	0.73
SD	0.59	0.02	0.40	0.05	0.33	0.03	0.20	0.03	0.30	0.02	0.30	0.03
Min	4.76	0.66	5.29	0.69	5.24	0.70	5.32	0.70	5.32	0.70	5.32	0.70
Max	6.76	0.74	6.90	0.85	6.37	0.82	5.95	0.83	6.45	0.75	6.45	0.78
Mean Po/Pb ratio org/inorg	8.3		7.9		7.7		7.7		7.9		7.8	

**Table 5:** Polonium/lead (^210^Po/^210^Pb) activity concentrations (mBq/g) and activity concentration ratios in the total aerosol samples collected at two sampling sites.

Sampling Date	Remote Site			Urban Site		
	^210^Po	^210^Pb	Po/Pb	^210^Po	^210^Pb	Po/Pb
	mBq/g	mBq/g	Ratio	mBq/g	mBq/g	Ratio
3 February 2019	464.8	315.2	1.5	530.8	373.0	1.4
3 March 2019	552.9	372.2	1.5	482.0	375.3	1.3
7 April 2019	567.7	298.8	1.9	492.3	352.6	1.4
5 May 2019	584.5	317.1	1.8	539.1	378.0	1.4
2 June 2019	469.2	327.4	1.4	500.2	361.4	1.4
7 July 2019	611.4	364.2	1.7	489.9	382.1	1.3
4 August 2019	557.9	313.8	1.8	548.1	382.6	1.4
1 September 2019	455.7	365.0	1.2	545.1	375.6	1.5
6 October 2019	594.7	310.8	1.9	499.3	363.0	1.4
3 November 2019	595.7	431.9	1.4	505.2	377.7	1.3
1 December 2019	562.1	469.0	1.2	458.6	339.2	1.4
5 January 2020	539.7	424.9	1.3	506.0	359.7	1.4
19 January 2020	550.4	371.4	1.5	570.3	400.0	1.4
2 February 2020	528.6	375.4	1.4	620.4	421.7	1.5
Mean			1.6			1.4
SD			0.2			0.1
Min			1.2			1.3
Max			1.9			1.5

**Table 6:** Polonium-210 and ^210^Pb activity concentrations (mBq/m^3^, or otherwise indicated) and ^210^Po/^210^Pb ratios in surface air at several locations around the world.

Location	Description of the Area	^210^Po (mBq/m^3^)	^210^Pb (mBq/m^3^)	^210^Po/^210^Pb	Reference
Kuwait (North of the country)	Desert	0.63 (0.09–1.48)	0.47 (0.37–0.58)	1.6 (1.2–1.9)	This study
Kuwait City	Urban	0.63 (0.15–1.41)	0.46 (0.98)	1.4 (1.3–1.5)	This study
Sacavém-Lisbon Portugal	Urban, near the Atlantic coast	0.031 ± 0.038	0.204 ± 0.107	0.19 ± 0.19 (0.03–0.78)	[23]
Kanpur, India	Urban	0.094 (0.0–0.28)	1.8 ± 1.1	0.079 (0.002–0.229)	[7]
Detroit, Michigan USA	Urban	0.072 (BDL-0.118) ^a^	0.30–4.22	0.075 (0.0–0.21)	[50]
Malaga, Spain	Urban, inland	0.045–0.070	0.51	0.11–0.13	[51]
Boulder, Colorado, USA	Urban, inland	0.002–0.052	0.15–0.78	0.01–0.06	[4]
Alaska, USA	Cold Desert	0.0–0.152	0.22–1.02	0.0–0.177	[52]
Lodz, Poland	Urban smelters nearby	0.067	0.556	0.12	[53]
Portugal	Smoke from forest fires Reference area	7255 ± 285 Bq/kg (dw) 111 ± 7 Bq/kg (dw)	2070 ± 88 Bq/kg (dw) 5895 ± 218 Bq/kg (dw)	3.5 (1–12) 0.018	[9,12]
Taranto, Italy	Area near iron and steel making factory	Nov 0.104 ± 0.060 May 0.291 ± 0.140	0.623 ± 0.325 0.812 ± 0.179	0.168 ± 0.037 (0.118–0.231) 0.343 ± 0.097 (0.231–0.543)	[54]
Worldwide average	Data compilation	0.050	0.500	0.1	[16]

^a^ BDL = below detection limit.

## Data Availability

Data are archived at KISR.
